# High-resolution texture/friction dataset: Characterizing asphalt pavements and aggregate mosaics at varied polishing stages for measured friction analysis

**DOI:** 10.1016/j.dib.2023.109384

**Published:** 2023-07-06

**Authors:** Malal Kane

**Affiliations:** Univ Gustave Eiffel, Campus de Nantes, AME/EASE, Allée des ponts et chaussées, 44340 Bouguenais, France

**Keywords:** Texture, Friction, Asphalt pavement, Aggregate mosaic, Polishing

## Abstract

This database comprises high-resolution topographies of 30 distinct surface textures, derived from five different types of aggregates. Each topography is in “txt” format and is composed of 15 parallel profiles separated by 0.5 mm. Each profile has a length of 76 mm of 0.01 mm resolution. We created both asphalt and mosaic samples for each aggregate type, resulting in a total of 10 sample variations (5 asphalts + 5 mosaics). Each of the 10 samples underwent three levels of polishing, yielding three distinct states of roughness per sample and a total of 30 unique surface textures.

Polishing tests were conducted using the Wehner-Schulze machine, which features two stations: one for polishing and another for measuring friction. During the polishing process, a rotary disc with three rubber cones rolled on the specimen surface, applying a designated load. To expedite the polishing, a mixture of 5% quartz powder (< 0.06mm) in 95% water was sprinkled onto the specimen. The polishing was performed on a ring, with the machine programmable to stop after a specific number of rotations.

Once the specimen was polished, it was manually transferred to the friction-measuring station, equipped with a rotary disc holding three small rubber pads arranged at 120°. Each rubber pad had an average length of approximately 30 mm, a width of 15 mm, and a thickness of 4 mm, loaded at 56N (refer to Figure 3). To measure friction, the disc was launched and accelerated to a speed of 100 km/h at its circumference. At 90 km/h, water was sprayed onto the specimen surface. Upon reaching 100 km/h, the motor stopped, and the disc descended until the rubber pads contacted the specimen surface. The rotation ceased due to the friction between the rubber pads and the specimen, and the resulting friction-speed curve was recorded. The analysis focused on the friction value at 60 km/h.

**Specifications Table**

**Table:** 

Subject	Civil and Structural Engineering
Specific subject area	Road engineering
Type of data	The data are composed of profiles in “txt” files and an Excel table giving the friction values. the ``.txt'' files can be opened directly by any software able to read ``.txt'' files.
How data were acquired	The dataset is provided in “.txt” format and organized into the following classification structure: There are five main directories, each containing two subdirectories representing a specific type of aggregate (one for mosaic and one for asphalt). Within each of these subdirectories, there are three additional directories corresponding to different levels of polishing (0, 90000, and 180000 polishing cycles). Within these polishing directories, you will find 15 profiles presented as individual “.txt” files. Additionally, there is a separate Excel file specifically for friction measurements, which includes the Coefficient of Friction (CoF) values obtained for each of the asphalt and mosaic samples.
Data format	Raw
Parameters for data collection	Each topography is in “txt” format and is composed of 15 parallel profiles separated by 0.5 mm. Each profile has a length of 76 mm. Every two points of the profile are separated by 0.01 mm. The friction file is an Excel file. It includes the Coefficient of Friction (CoF) values obtained from Wehner-shulz friction measurement for each of the asphalt and mosaic samples.
Description of data collection	The texture of each asphalt pavement and aggregate mosaic was captured using a laser scanning profilometer before and after 90,000 and 180,000 cycles of polishing. The measurement area is situated within the polished ring of the samples. Topography data were acquired through 15 parallel profiles, each 76 mm in length, with a sampling interval of 0.01 mm and separated by 0.5 mm intervals. The data is stored in “.txt” format and organized according to the directory structure described in the previous section. Friction data were obtained using the friction head of the Wehner-Schulz machine. To measure friction, the disc was launched and accelerated to a speed of 100 km/h at its circumference. At 90 km/h, water was sprayed onto the specimen surface. Once the speed reached 100 km/h, the motor was stopped, and the disc descended until the rubber pads contacted the specimen surface. The rotation ceased due to the friction between the rubber pads and the specimen surface, and the resulting friction-speed curve was recorded. The friction value considered for analysis is at 60 km/h.
Data source location	Institution: Université Gustave Eiffel City/Town/Region: 44340 Bouguenais Country: France
Data accessibility	Kane, Malal (2023), “High-Resolution Texture/Friction Dataset: Characterizing Asphalt Pavements and Aggregate Mosaics at Varied Polishing Stages for Measured Friction Analysis”, Mendeley Data, V1, doi: 10.17632/kkcztnxzph.1 Accessible via the following link: https://data.mendeley.com/datasets/kkcztnxzph/1

## Value of the Data

These data hold significant value for the road scientific community engaged in research on the relationship between pavement texture and friction. Indeed, they can be used to initiate investigations into the determination of suitable pavement texture parameters to represent its friction capacity. This is still a big debate in the community.

It marks the first instance where the community can freely access high-resolution data on pavement textures, showcasing their evolution when subjected to the Wehner-Schulz machine's polishing process, along with the corresponding friction measurements.

In summary, this dataset offers:

• Access to high-resolution pavement textures captured at a resolution of 10 microns, utilizing asphalt pavements and aggregate mosaics of various aggregates and representing different levels of polishing.
• The accompanying measured friction values obtained from these asphalt and mosaic samples at each respective polishing level.

## 1 Data Description

The texture of each asphalt pavement and aggregate mosaic was captured using a laser scanning profilometer before and after undergoing 90,000 and 180,000 cycles of polishing. The measurements were conducted within the polished ring area of the samples. Topography data were obtained through 15 parallel profiles, each measuring 76 mm in length, with a sampling interval of 0.01 mm and separated by 0.5 mm intervals.

The dataset is provided in “.txt” format, openable with any software capable to read a txt file, and organized in the following structure: There are five main directories, each containing two subdirectories representing specific aggregate types (one for mosaic and one for asphalt). Within each subdirectory, three additional directories exist, corresponding to different levels of polishing (0, 90,000, and 180,000 polishing cycles). Within these polishing directories, individual “.txt” files present 15 profiles.

Additionally, a separate file is dedicated to friction measurements, encompassing the Coefficient of Friction (CoF) values obtained for both asphalt and mosaic samples.

Friction data were acquired using the friction head of the Wehner-Schulz machine. To measure friction, the disc was launched and accelerated to a speed of 100 km/h at its circumference. At 90 km/h, water was sprayed onto the specimen surface. When the speed reached 100 km/h, the motor was stopped, and the disc descended until the rubber pads contacted the specimen surface. The rotation ceased due to the friction between the rubber pads and the specimen surface, allowing the recording of the resulting friction-speed curve. The friction value considered for analysis is at 60 km/h.

## 2 Experimental Design, Materials and Methods

### 2.1 Surfaces preparation

**Fig. 1:**
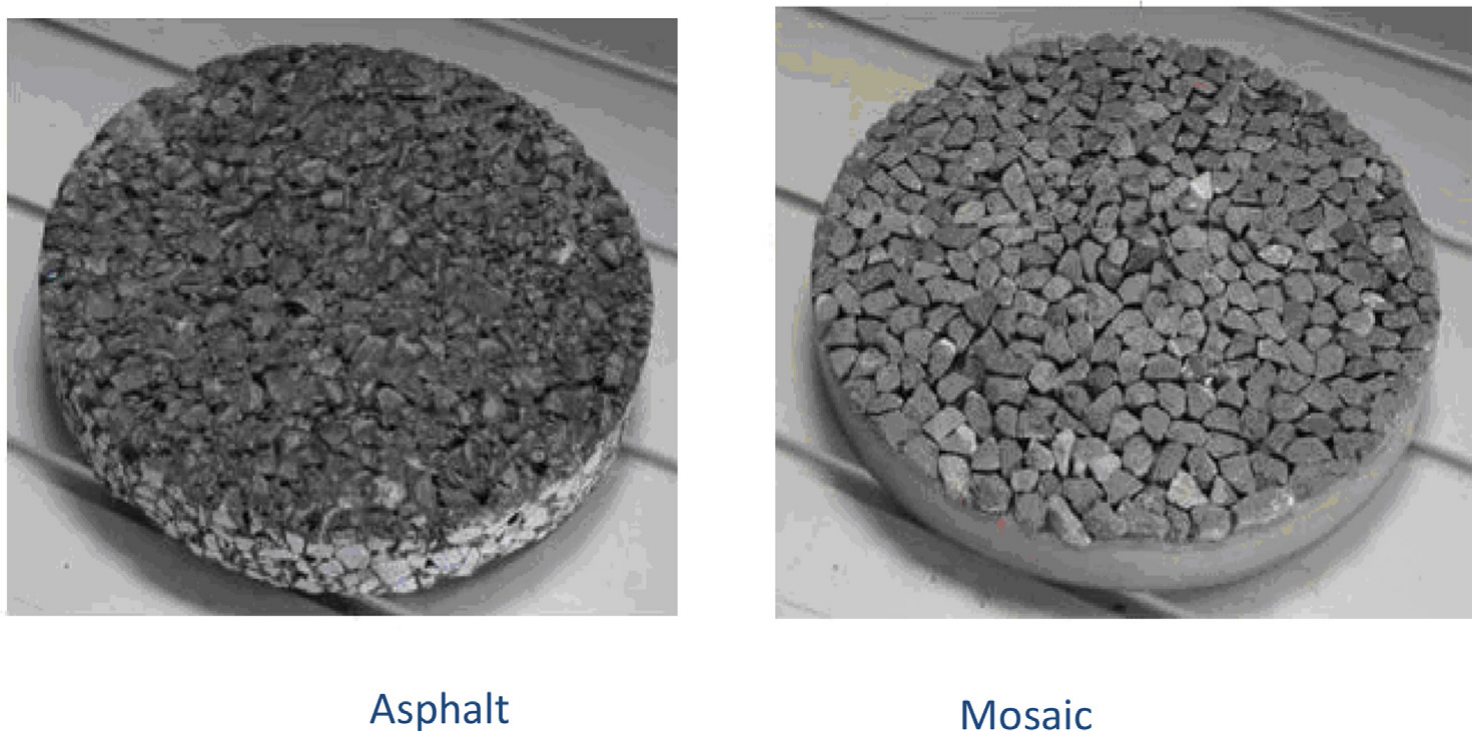
The left side shows a core extracted from laboratory-manufactured asphalt slabs, while the right side displays mosaics of aggregates prepared from aggregates of size.

**Fig. 2:**
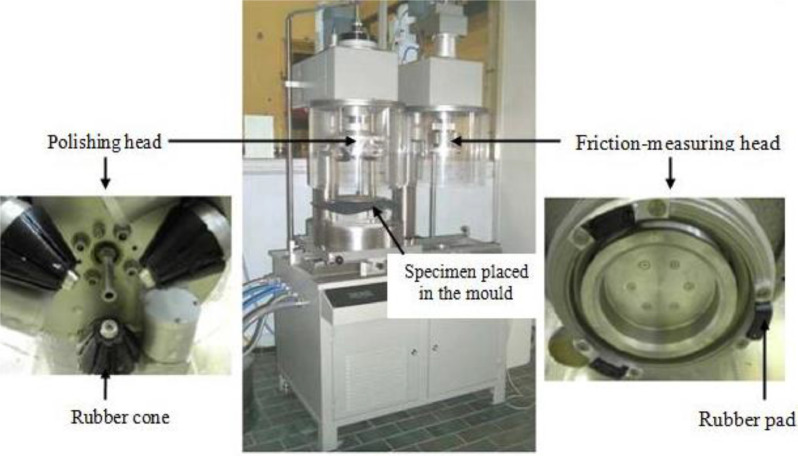
The Wehner-Schulze (WS) machine used for surface polishing and friction measurement. The machine comprises two stations, one for polishing and another for measuring friction.

The texture data is composed of 30 surfaces obtained from 5 different aggregate types. For a comprehensive evaluation of surface texture, asphalt and mosaic samples (Fig. 1) were created for each aggregate type, resulting in a total of 10 sample types. Each sample underwent three levels of polishing, resulting in three distinct roughness states and 30 unique surface textures. Polishing was conducted using the Wehner-Schulze machine (Fig. 2), which consists of two stations: one for polishing and the other for measuring friction. A rotary disc with three rubber cones rolled on the specimen surface with a designated load, and a mixture of 5% quartz powder in water was used to speed up the process. The study involved three types of aggregates, two types of samples, and three levels of polishing [1,2].

### 2.2 Friction measurements

After polishing, the specimens were moved to the friction-measuring station of the Wehner-Schulze machine (Fig. 2). The station had a rotary disc with three small rubber pads arranged at 120°. The pads, curved with specific dimensions, were loaded at a designated force. Friction was measured by launching the disc and accelerating it to a speed of 100 km/h while spraying water on the specimen surface at 90 km/h. The rotation stopped when the rubber pads contacted the specimen surface, and the resulting friction-speed curve was recorded. The analysis focused on the friction value at 60 km/h [1,2].

Acquiring Specimen Textures:

The textures are captured using a high-resolution laser profilometer measured from "STIL" named “Stil Micrometer”. The characteristics of the profilometer are given below:

• Measurement range: 350 μm
• Measurement error: ± 0.03 µm
• Lateral resolution: 3 µm

**Fig. 3:**
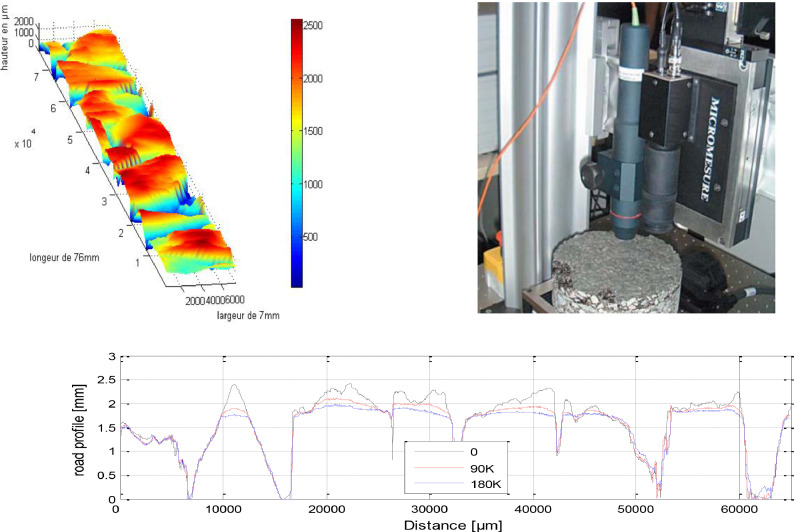
Example of recorded topography of one of the studied surfaces. Up-right: The profilometer, Up-Left: 3D View, Bottom: A profile at three levels of polishing.

The texture of each of the 30 surfaces with a precision of 10 µm (Fig. 3). The original texture data consisted of 15 parallel profiles, each measuring 76 mm in length and spaced at 0.5 mm intervals. These profiles were sampled at a resolution of 10 µm, providing a detailed representation of the surface texture. The profilometer was positioned perpendicular to the specimen surface and scanned along the length of each profile to capture the texture data [1,2].

## Ethics Statements

Nothing to declare.

## CRediT authorship contribution statement

**Malal Kane:** Methodology, Data curation, Investigation, Validation.

## Declaration of Competing Interest

The author declares that he has no known competing financial interests or personal relationships which have or could be perceived to have influenced the work reported in this article.

## Data Availability

High-Resolution Texture/Friction Dataset: Characterizing Asphalt Pavements and Aggregate Mosaics at Varied Polishing Stages for Measured Friction Analysis (Original data) (Mendeley Data).
